# Methods and tools used to assess patient experience with healthcare services in prisons: a scoping review

**DOI:** 10.1093/intqhc/mzag005

**Published:** 2026-01-29

**Authors:** Katherine E McLeod, Amie Hilditch, Amanda Butler, Rith Bal, Fiona G Kouyoumdjian

**Affiliations:** Department of Family Medicine, McMaster University, Hamilton, ON, Canada; Department of Family Medicine, McMaster University, Hamilton, ON, Canada; School of Criminology, Simon Fraser University, Burnaby, BC, Canada; School of Interdisciplinary Science, McMaster University, Hamilton, ON, Canada; Department of Family Medicine, McMaster University, Hamilton, ON, Canada

**Keywords:** Prison Health Service, prisons, scoping review, health services research, patient experience

## Abstract

**Background:**

Measures of patient experience of healthcare services are important to improving quality of care and health outcomes. In correctional facilities, healthcare is shaped by key features of the environment, including security constraints and uncertain lengths of stay. In this scoping review, we aimed to map the existing literature and identify knowledge gaps to inform efforts to implement and enhance measures of patient experience in correctional settings.

**Methods:**

We conducted a search of health sciences databases and grey literature for original research articles on 6 December 2024. We included studies in which people currently incarcerated in a correctional facility were asked about their experiences of healthcare received in custody. Two people independently assessed all titles and abstracts, reviewed full texts, and completed data extraction. We assessed how measures of patient experience in each study aligned with the Picker Principles of Patient-Centred Care.

**Results:**

Our search identified 710 unique records, and we included 44 articles reporting on 42 studies in this review. Among included studies, 21 (52%) assessed patient experience using surveys, 18 (43%) used qualitative methods, and 3 (5%) used a combination. No studies reported using a survey that had been previously validated for healthcare delivered in a correctional facility. Questions about communication with healthcare staff and access to services were more common than inquiry into perceptions of respect, empathy, and autonomy. No study asked about the involvement of family and friends in care. We found gaps in the geographic representation of studies and in reporting considerations of confidentiality and compensation for participants.

**Conclusions:**

There is a need for validated, context-specific tools to measure patient experiences of healthcare in custody, with attention to autonomy, respect, and relational aspects of care. Addressing current gaps will support person-centred, equitable healthcare in correctional settings and support quality improvement and accountability.

## Introduction

Patient experience is a key aspect of healthcare quality and enhancing patient experience of care is one of the core pillars of improving health systems outlined in the Quintuple Aim [1]. Positive patient experiences are associated with higher levels of patient safety and clinical effectiveness, greater adherence to treatment, better health outcomes, and reduced burden on healthcare resources [2]. In correctional facilities, where delivery of healthcare services is shaped by structural and security constraints unique to the carceral environment, understanding and measuring patient experience is especially important.

People who experience incarceration have a higher burden of mental and physical health conditions and often have complex healthcare needs [3, 4]. They also face systemic barriers to accessing healthcare both in custody and in the community, including stigma based on their history of criminalization [5–7]. While in community healthcare settings measures of patient-reported experience or satisfaction with healthcare are used, and in some cases required [8, 9], to monitor quality of care, they are uncommon in the context of correctional facilities. Frameworks of patient-centred care, such as the Picker Principles of Person-Centred Care [10] highlight key dimensions of person-centred healthcare including involvement in decision-making, respect, communication, and access to care. In prisons, these principles may be in tension with policies and priorities of security and control. Therefore, tools used in community settings to measure patient experience may not capture the distinct realities of people receiving care in the carceral context such as lack of privacy or confidentiality, altered access to medications, and interactions with healthcare that are compelled by procedure rather than choice [11–13]. The carceral environment also presents unique challenges to data collection such as access to potential participants and perceptions of confidentiality or coercion [14].

There is a need for robust, context-specific methods and tools for measuring patient experience of healthcare in custody in order to improve healthcare services and address health inequities. In this context, the objective of this scoping review is to map the existing literature and to identify and analyse knowledge gaps related to measuring patient-reported experience of healthcare services in correctional facilities.

## Materials and methods

We conducted this scoping review following the JBI guidelines [15] and we report methods in alignment with the Preferred Reporting Items for Systematic Reviews and Meta-analysis extension for scoping review checklist [16]. This review was guided by the research question: how have studies reported processes and tools used to measure patient experience of healthcare services in correctional facilities?

### Eligibility criteria

We included full-text, original research articles which described methods of understanding or measuring patient experiences of healthcare services delivered in correctional facilities. Specifically, study participants had to be incarcerated at the time of the study in facilities related to the administration of criminal justice (i.e. we excluded studies with people previously incarcerated, or currently in secure mental health facilities, or immigration detention centres, or prisoners of war). We considered ‘healthcare services’ to be the fundamental functions of healthcare services including testing, diagnosis, condition management, and treatment. We excluded studies investigating ancillary programmes (e.g. stress management or domestic violence workshops) as these are outside the scope of fundamental functions of healthcare services, and we also excluded studies in which the primary focus was describing experiences of a particular illness or condition rather than the healthcare received.

### Information sources

We developed and refined our search strategy in consultation with a health sciences research librarian. We used index records of relevant articles identified in an initial search of MEDLINE and Embase to develop a full search strategy for MEDLINE (Appendix 1). The search strategy, including keywords and index terms, was translated to Embase, Emcare, PsycInfo, and Web of Science Core Collection. We also searched the first 10 pages of Google Scholar and the reference lists of all articles included in our study for relevant studies. We included studies published in any language, though we used only English-language search terms. Articles not in English were translated using Google Translate, which has been shown to be accurate for the purpose of extracting data for systematic reviews [17]. There were no date limitations imposed on our search. The most recent search was conducted on 6 December 2024.

### Selection of articles

We uploaded all identified records into Covidence [18], a web-based collaboration platform designed to streamline ­review processes. After duplicates were removed, two project team members independently assessed all titles and abstracts for eligibility using predetermined inclusion criteria. Articles included for full review were independently assessed by two project team members. At both stages, reviewers discussed any disagreements until consensus was reached.

### Data charting

To collect data, a charting form was developed by the research team. Two team members independently applied the charting form to the same five articles and then met to discuss and refine the charting form prior to its use on the full data set, as recommended by Levac *et al*. [19]. Two reviewers independently extracted data from each included study. Consistent with standards for scoping reviews, we did not conduct any quality appraisals [16]. We collected data on study location, number/type of correctional facilities, participant characteristics, the specific healthcare services studied, study design (including recruitment and sampling methods, data collection, compensation, and confidentiality considerations), the study’s data collection tool(s), data collected, major topics of inquiry, study limitations reported by authors, and intended application of study findings.

To assess the aspects of healthcare explored in studies of patient experience, we examined topics of inquiry presented in data tools (e.g. interview scripts, survey questions) or, when tools were not provided, questions investigated as reported by study authors. We categorized topics using the eight Picker Principles of Person-Centred Care [10]. These principles set out a framework for high-quality person-centred care across patient pathways: (i) fast access to reliable healthcare advice; (ii) effective treatment by trusted professionals; (iii) continuity of care and smooth transitions; (iv) involvement and support for family and carers; (v) clear information, communication, and support for self-care; (vi) involvement in decisions and respect for preferences; (vi) emotional support, empathy, and respect; (vii) attention to physical environment and needs. Examples of questions categorized within each principle are provided in Appendix 2.

## Findings

**Figure 1:**
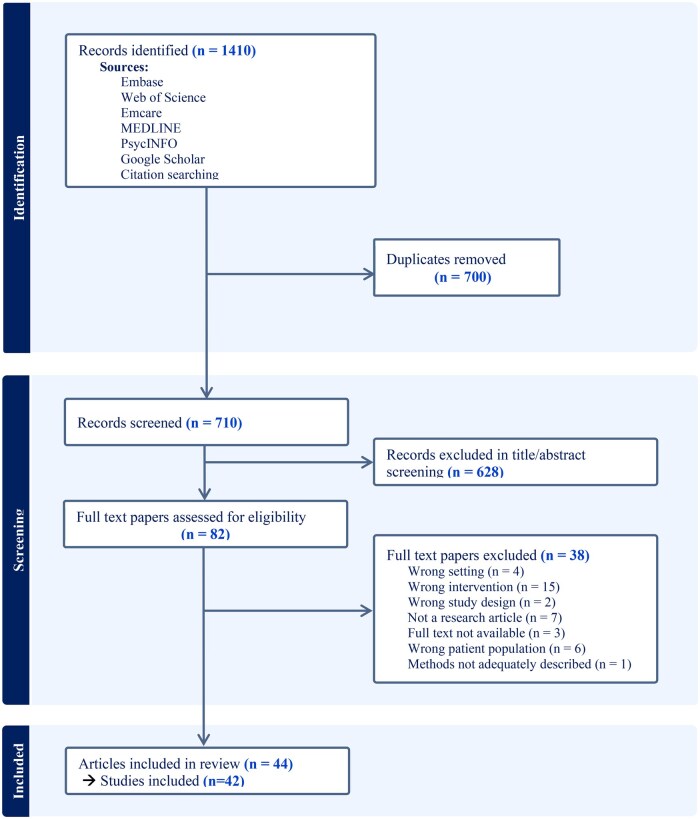
PRISMA flow diagram of study selection process.

After duplicates were removed, our search identified 710 records for title and abstract screening. Of these, 82 articles were advanced to full text review. One article in German [20] was translated to English using Google Translate. We found two papers reporting the same findings from the same study and published in the same year. We retained the original research article for data extraction [21] and excluded the shorter summary article [22]. Finally, 44 papers reporting on 42 studies were included in this study (Fig. 1).

### Study design

A summary of included studies is provided in Appendix 3. Studies were conducted in 16 countries, with more than half conducted in one of three countries: the USA (*n* = 10, 24%) [21, 23–31], UK (*n* = 8, 19%) [11, 32–38], and Australia (*n* = 6, 14%) [39–45]. Three studies were conducted in each of Canada [46–48], and Spain [49–51], two studies were conducted in South Africa [52, 53] and one study was conducted in each of China [54], Germany [20], Greece [55], Iran [56], Lebanon [57], Norway [58], Poland [59], Switzerland [60, 61], Taiwan [62], and Thailand [63]. Surveys were the most common method of data collection and were used in 24 studies [20, 23, 25–30, 36, 37, 39, 40, 45, 48–52, 54, 55, 57–59, 62] including three studies which used a combination of survey and qualitative methods [20, 48, 52]. Eighteen studies used qualitative methods, including interviews (*n* = 10) [21, 24, 31–34, 42–44, 56, 61], focus groups (*n* = 5) [38, 41, 46, 47, 53], or a combination of both (*n* = 3) [11, 35, 63]. Studies measured experiences of healthcare generally (*n* = 19) [11, 20, 24, 26, 29, 31, 33, 35–41, 45, 46, 53, 58–63], mental healthcare (*n* = 7) [25, 27, 28, 30, 34, 54, 55], reproductive healthcare including pregnancy care (*n* = 4) [14, 21, 47, 56], substance use and addictions care (*n* = 4) [44, 48, 51, 57], specialist care (*n* = 2) [26, 37], care for hepatitis C (HCV; *n* = 2) [42, 43, 50], and testing for sexually transmitted and blood-borne infections (*n* = 2) [42, 43, 52]. Additionally, one focus group study examined methods of gathering patient feedback on experiences of healthcare in custody used in two English prisons [33], and one study examined experiences of care among transgender people taking hormones in custody [23]. Nine studies examined the acceptability of telemedicine or telepsychiatry [20, 25–28, 49, 50, 54, 55]. The midpoint for data collection for just under half of studies (17/42, 40%) was within the last 10 years (between 2015 and 2021) [20, 24, 33, 38, 41–43, 45–50, 54–57, 59]. Twelve studies did not report study dates [21, 23, 26, 27, 29, 31, 32, 34, 35, 37, 53, 63].

### Participants

The number of people included in each study ranged from 4 [34] to 4887 [36] [median 75, interquartile range (IQR) 25–200]. One study did not specify number of participants [53]. Eighteen studies examined experiences of men in custody [23, 25, 27, 30, 34, 37, 38, 42, 43, 45, 49–55, 57, 62], eight studies explored experiences of women [21, 31, 32, 35, 46, 47, 56, 63], and the remaining 16 included both men and women with the proportion of women ranging from 3.3% [39] to 66.7% [33] (median 8.6%, IQR 0%–30.8%). One study reported on the healthcare experiences of transgender people in custody [23].

Three Australian studies [40, 42–44] reported compensating participants $10 into their prison bank account, one US study reported compensating $20 [21], and one Canadian study reported giving participants edible items from the canteen worth $10 [46]. Nine studies reported that compensation was not provided, with two Canadian studies [47, 48] and one US study [31] specified that compensation was not allowed by correctional authority policy. The remaining 28 studies did not report whether people participating in the research received any compensation.

### Confidentiality

In 17 studies, data collection was conducted by an external researcher. For 11 studies using surveys, data collection was facilitated or collected by staff [28–30, 37, 39, 40, 45, 51, 57–59]; including two studies in which healthcare staff collected data in facilities in which they did not routinely practice [39, 64], one study where staff were deployed from a correctional services central office [29] and one study in which surveys were distributed by correctional staff [59]. Fourteen studies did not specify who collected data [20, 21, 23, 25–27, 36, 44, 49, 52, 54, 55, 62, 63]. Nine studies (seven qualitative and two survey) reported that data collection took place in a private space within the prison, such as clinic rooms, or specified that staff were not present [11, 23, 24, 31, 35, 39, 42, 43, 56, 60, 61], including one study reporting that a correctional officer was positioned outside the room but in line-of-sight [39].

Three studies reported evaluation processes that were not confidential: one conducting focus groups in the presence of prison officers [41], another reported that staff wrote participants names across questionnaires as they were distributed [28], and one reported conducting interviews in the presence of the person’s mental healthcare clinician and submitting transcripts to the prison’s security department [34]. Fifteen studies did not report on how confidentiality was addressed [21, 25–27, 32, 38, 46, 49, 52–54, 56–58, 63].

### Patient experience measurement

A wide variety of tools was used among the 24 studies that reporting using surveys (including three studies that used a combination of survey and qualitative methods). Four studies [25, 36, 39, 45] used tools that had been previously developed specifically for use in custody, including the Measuring the Quality of Prison Life Instrument, [36] but none reported whether or how these tools had been validated in the carceral context. Six studies reported using a survey tool developed for or as part of the study based on the literature, community surveys, or patient complaints [20, 23, 26, 28, 29, 54] and eight used or adapted tools from community settings including for psychiatric outpatient care [27, 30, 37, 51, 55, 57, 58, 62]. Of these, three studies reported piloting the survey with a small number (10 or fewer) of people in custody [20, 37, 55] and one engaging experts regarding surface validity but did not describe the five experts [62]. The remaining six studies did not report how surveys were developed.

Table 1 provides a summary of how measures of patient experience reported in each study align with the Picker Principles of Patient Centred Care. No studies asked any questions regarding the involvement and support of family members and carers. Studies most frequently included questions within the domain of clear information, communication and self-care (*n* = 17), followed by access to healthcare services (*n* = 16), and effective treatment by trusted professionals (*n* = 15), attention to the physical environment (*n* = 9), patient’s perceptions of emotional empathy and respect from staff (*n* = 9), patient’s involvement in decision-making (*n* = 6), and continuity of care (*n* = 4). Other common measures of patient experience included overall satisfaction with care (*n* = 19), asking people to compare services in custody to services in the community (*n* = 7) [11, 25, 35, 40, 41, 46, 50] and how people gained knowledge of how to access healthcare services in custody (*n* = 3) [11, 39, 41]. Additionally, 24 studies collected demographic information, [11, 20, 21, 24, 36, 40–44, 46–48, 50–62], 20 asked about health history or previous use of healthcare [21, 23, 24, 28, 31, 33, 37, 39, 40, 42, 43, 46–49, 54, 57–62], and 14 collected custody data (such as sentence length) [11, 20, 36, 40, 41, 44, 48, 52, 55, 56, 58–62].

**Table 1:** Areas of inquiry represented in study instruments as reported in studies of patient experiences of healthcare services delivered in correctional facilities.

	Picker Principles of Person-centred Care								Other question types	
Study	Fast access to reliable healthcare advice	Effective treatment by trusted professionals	Continuity of care and smooth transitions	Involvement and support for families and carers	Clear information, communication and support for self-care	Involvement in decisions and respect for preferences	Emotional empathy and respect	Attention to physical and environmental needs	Overall satisfaction^a^	General (qualitative methods)^b^
Abbott 2023 [32]										x
Alirezaei 2022 [56]										x
Barling 2005 [39]	x				x	x	x		x	
Besney 2018 [46]	x								x	x
Bjorngaard 2009 [58]		x	x		x	x			x	
Butler 2003 [40]	x	x	x				x		x	
Capon 2020 [41]	x				x		x			
Cheng 2018 [54]							x	x	x	
Colombo 2022 [20]	x	x			x			x	x	
Condon 2007 [11]										x
Cuadrado 2021 [49]	x	x			x			x	x	
Galli 2019 [61] Heidari 2017 [60]										x
Hankins 2022 [33]										x
Hsieh 2022 [62]	x				x		x	x	x	
Jaffer 2016 [23]	x									x
Jimenez-Galan 2019 [50]		x	x		x			x	x	
Jordan 2012 [34]										x
Kanbergs 2019 [24]	x									x
Karachaliou 2024 [55]	x	x	x		x		x		x	
Khalaf 2019 [57]	x	x			x					
Lafferty 2022 [42] Lafferty 2023 [43]										x
Liauw 2021 [47]										x
Magaletta 2000 [25]		x						x	x	
Marco 2013 [51]						x			x	
Mekhjian 1999 [26]		x			x			x		
Morgan 2008 [27]									x	
Motshabi 2011 [52]	x	x			x	x			x	
Myers 2006 [28]		x			x			x	x	
Plugge 2008 [35]										x
Rogalska 2022 [59]	x									
Ross 2011 [36]	x				x		x			
Russell 2022 [48]										x
Sawasdipanich 2018 [63]										x
Schroeder 2005 [21]										x
Sifunda 2006 [53]										x
Sindicich 2016 [44]										x
Tanguay 2014 [29]	x	x			x		x		x	
Tucker 2005 [37]		x			x				x	
Way 2007 [30]	x	x			x	x	x		x	
Williams 2022 [38]										x
Wong 2018 [45]		x			x	x		x	x	
Young 2000 [31]										x

a Measures of overall satisfaction including satisfaction rating scales and questions related to recommending services to others, or willingness to use services again.

b General included open-ended questions asked in interviews and focus groups such as ‘what was your experience with healthcare services’.

## Discussion

### Statement of principle findings

In this global scoping review, we identified 44 papers describing 42 studies of patient-reported experiences of healthcare received in correctional facilities. Surveys were used to understand patient experience in 21 studies, 18 studies used qualitative methods (focus groups and/or interviews), and three studies used a combination of survey and qualitative methods.

### Interpretation within the context of the wider literature

Though surveys were used in just over half of studies (*n* = 24, including three studies which used survey and qualitative methods), there was a lack of tools tailored to, or validated in, correctional facilities. Surveys designed for other settings may result in inaccurate or incomplete measures of patient experience by overlooking elements of the carceral environment which shape healthcare services. For example, correctional officers are directly responsible for security and control, but often participate in providing or facilitating access to healthcare [65]. There is a need for validated patient experience measures within correctional institutions to support benchmarking and quality improvement. Qualitative methods may offer more opportunity to capture complexity, but have other limitations; for example, they traditionally require more time and resources, as well as research expertise to collect and analyse data [66]. Additionally, like studies of qualitative methods in prisons more generally, this review highlighted challenges to qualitative methods of patient experience data collection in correctional facilities including lack of privacy and confidentiality and the involvement of correctional and healthcare staff in recruitment [14].

In this review, questions about clear information, communication, and self-care were explored in 17 studies, but questions about respect (*n* = 9) and agency or involvement in care (*n* = 6) were less commonly explored. Qualitative studies have described prevalent negative interpersonal relationships with healthcare staff in which people in custody feeling they are being treated disrespectfully and not being listened to by providers [11, 67, 68]. Some have also found that people in custody expect lower-quality care from healthcare providers in prison than from community providers. These findings highlight how structural stigma (defined as conditions, norms, and policies which constrain resources, opportunities, and wellbeing of people being stigmatized [69]) and the inherent imbalance of power between providers and people in custody may shape experiences of care and health outcomes. This suggests that though it has been limited in published studies to-date, exploring respect and control or autonomy in decision-making may be particularly important to understanding and improving experiences of care in custody. Questions about satisfaction with services, an outcome measure of patient experience [70] were the most common type of question in included studies (*n* = 19) and 15 studies explored the domain of effective treatment by trusted professionals. However, outcome measures may be complicated to interpret in this setting as satisfaction is influenced by other factors including need, and expectations of care [70]; previous qualitative studies have reported that people in custody may have lower expectations of healthcare in custody compared to services in the community [67].

No studies included a measure of the involvement of support or families in care, despite being recognized as an essential component of high-quality person-centred healthcare [10]. In a US study, nurses reported that institutional policies restrict or prohibit sharing information with families, but that opportunities to engage with families as part of care would improve nursing practice and patient outcomes [71]. Beyond family, there exist supportive relationships between people in custody including informal supports, like friendships, and formal peer support roles [72]. The absence of any recognition of the role of personal support in experiencing healthcare needs or services in custody is a significant limitation in work to assess or improve patient-centred care.

### Strengths and limitations

More than half of studies in this review came from three countries (USA, UK, and Australia). The lack of studies from some regions may be partially explained by our use of English-language search terms which may have missed studies in other languages. However, this pattern is not unique to our review, other reviews of studies of patient experience measures in emergency departments [73] for people experiencing homelessness [74] and patient satisfaction with mental health services in prisons [75] also found that most studies came from these three countries, with more than half originating in the USA, suggesting that this concentration could also reflect interest or other factors in the healthcare and/or research environments.

A limitation is that our search and screening process included studies with an explicit reference to understanding patient experiences or satisfaction with healthcare services in custody in the title and/or abstract. This may have missed studies including patient experience measures as a component of other work (e.g. studies seeking to understanding experiences of disease in custody). As noted, we worked with a research librarian to obtain a feasible number of records enriched for relevance. We mitigated this limitation by hand-searching reference lists of included articles for additional relevant studies.

### Implications for policy, practice, and research

This review identified opportunities for enhanced practices and in research engaging people in custody in patient experience work, including attention to potential risks and benefits. Researchers have a responsibility to protect the rights and dignity of participants and to avoid doing harm [76, 77]. This review included examples of research in which participant identities and their responses were known to correctional and/or healthcare staff which may have affected people’s confidence and trust in the process, as well as the potential risk of negative impact on participants. A third of studies did not report any considerations related to confidentiality. Additionally, few studies reported on the provision of compensation for people participating in research. Low reporting on compensation has been found in other reviews of health research in carceral settings [4] and may be affected by institutional policy explicitly prohibiting compensation for research participants in some jurisdictions [78]. These gaps highlight opportunities to improve standards for, and reporting of, how people may be affected by participating in studies of patient experience measures in custody. As a minimum, to support transparency, researchers should provide detailed reporting of study protocols for protecting participant confidentiality, as well as identify any institutional policies impacting participant compensation.

The lack of tools measuring patient experiences of healthcare that are specific to, or validated in, carceral contexts has implications for healthcare policy and practice. Tools designed to understand healthcare in community settings may overlook distinct features of the prison environment and as a result data may not reflect the realities of experiences of care. Additionally, tools developed in community may not appropriately consider and address aspects of the collection of patient experience data in correctional institutions—such as lack of privacy, autonomy or power imbalances—which may have implications for the safety and wellbeing of participants and the validity of data collected. Patient experience measures used by studies in this review emphasized communication and information-sharing, while largely overlooking critical dimensions such as autonomy and shared decision-making, areas shaped by the constraints of incarceration. There is a need for tools and processes for measuring patient experience that are designed with the leadership of people with lived experience of incarceration to appropriately reflect and capture experiences of care in custody.

## Conclusion

Patient experience is a core measure of healthcare quality. This review highlighted critical gaps and opportunities for validated tools tailored to healthcare in the correctional environment. Addressing these gaps is essential to advancing person-centred, equitable healthcare in prisons, strengthening quality improvement efforts, enhancing accountability, and addressing health inequities faced by people experiencing incarceration.

## Data Availability

No new data were generated or analysed in support of this review.

## Appendix 1 Search strategy.

OVID Medline Epub Ahead of Print, In-Process & Other Non-Indexed Citations, Ovid MEDLINE(R) Daily and Ovid MEDLINE(R) 1946 to Present

**Table:**

1	Prisoners/
2	correctional facilities/or jails/or prisons/
3	(penitentiar* or prison* or correctional facilit* or correctional system* or correctional centre* or correctional center* or jail* or penal detention or gaol* or “deprived of freedom” or correctional institution* or “deprived of liberty” or criminal justice or justice facilit* or inmate*).kw, tw.
4	or/1–3
5	patient satisfaction/or patient preference/
6	consumer satisfaction/
7	(patient* experience* or client* experience* or patient* satisfaction or client* satisfaction or patient* feedback or client* feedback or satisfaction survey* or healthcare survey* or health care survey* or experience survey* or feedback survey* or patient* questionnaire* or client questionnaire* or healthcare questionnaire* or health care questionnaire* or patient satisfaction tool* or client satisfaction tool* or patient* perspective* or client* perspective* or service user perspective* or service user feedback or service user experience*).mp.
8	or/5–7
9	4 and 8

## Appendix 2 Examples of data collection questions and statements categorized by the Picker Principles of Person-centred Care.

**Table:**

Picker Principles of Person-centred Care	Examples from data collection instruments
Fast access to reliable healthcare advice	If I have a health problem, I can easily see a health professional [40] How easy is it to get health care in prison? [41] I felt the timeliness of consultation was acceptable [49]
Effective treatment by trusted professionals	Those who provide my health care are competent and well trained [40] Sometimes doctors make me wonder if their diagnosis is correct [62]
Continuity of care and smooth transitions	Did you feel the clinic staff worked as a team in your care and treatment? [41] Hospital and institutional physician communication [50]
Involvement and support for families and carers	None found
Clear information, communication and support for self-care	I was able to communicate what I wanted to say to the doctor [26] The doctor communicated with me effectively [26]
Involvement in decisions and respect for preferences	Mental Health staff involves me in decisions about my treatment [30] Felt they had the opportunity to be involved in decisions regarding treatment [39]
Emotional empathy and respect	Do you think you were treated with respect by the clinic staff? [41] The doctors here believe me when I tell them about my health problems [36]
Attention to physical and environmental needs	Were you satisfied with the audio quality of the telepsychiatry equipment? [54] I think my doctor’s office has everything needed to provide complete care [62] Was not concerned about being overheard by others [28] Not being in the same room with the doctor was a problem for me [26]
Overall satisfaction	Future use of service [20] I am satisfied with the healthcare I receive in prison [40] Overall I am satisfied with the service I received [49] Would you recommend TV treatment to other inmates? [25] How would you rate your overall experience with the Nurse Practitioner? [45]

## Appendix 3 Summary of characteristics of included studies.

**Table:**

**Author(s), (year), country**	Service	Study year(s)	Participants	Study type	Recruitment	Who collected data	Confidentiality	Data collection tool development
Abbott *et al*. 2023 [32], UK	Midwifery care	Not reported	22 adult women in 3 correctional facilities	Interview	Each prison maintained a list of pregnant women. Staff helped to locate women in the facility and facilitate recruitment	Researcher; a registered midwife with no previous experience of working in prisons	Not reported	Not reported
Alirezaei *et al*. 2022 [56], Iran	Prenatal care	2019–21	11 adult women in 1 correctional facility	Interview	Purposive sampling of pregnant women in custody	PhD student	Not reported	Not reported
Barling *et al*. 2005 [39], Australia	Healthcare services	2001 and 2004	203 adult men and 7 adult women across 24 correctional facilities	Verbally administered survey	Cluster random sampling of institutions then individuals selected from population lists	Registered nurse who did not practice in the correctional centre where they administered the survey	The survey was conducted in a room, with only the interviewer and the interviewee present, but with a correctional officer in line-of-sight	Developed by Justice Health
Besney *et al*. 2018 [46], Canada	A women’s health clinic	2014–5	11 adult women in 1 correctional facility	Focus groups	Self-selection. Potential participants were made aware of the focus groups at the time of their clinic assessment by a health care provider and through poster advertisement. Interested potential participants were approached by a nonstudy team member who ensured inclusion criteria were met and provided a study information sheet.	Two experienced qualitative interviewers	Not reported	Not reported
Bjorngaard *et al*. 2009 [58], Norway	Primary and specialist health care	2007	1096 men and 57 women in 29 correctional facilities	Survey	Two qualified psychiatric nurses contacted the prisoners, either individually in the cell, at mealtime, or at a meeting set up for the specific purpose of conducting the survey. In one small, remote prison the questionnaires were distributed and collected directly by the prison staff.	In 28 participating prisons surveys were distributed and collected by two psychiatric nurses with clinical prison experience; in one remote prison surveys were distributed and collection by prison staff	Not reported	Adapted a satisfaction scale developed and validated for use in psychiatric outpatient settings.
Butler 2003 [64], Australia	Healthcare services	2001	747 men and 167 women in 29 correctional facilities	Survey	Potential participants were called up to the interview area and given a full explanation of the project by a member of the interview team	Nursing staff within correctional health services (not in their place of work) including Indigenous nurses who screened Indigenous participants	Participants were told that information would be treated with the utmost confidentiality and that questionnaires would be marked with an anonymous identification code.	Not reported
Capon *et al*. 2020 [41], Australia	Healthcare services	2017	99 men and 29 women in 12 correctional facilities	Focus groups	Potential research participants were randomly selected from a patient list and through self-selection from promotional posters that were located in the health centres and on wings of the selected prisons several weeks prior to data collection.	Researchers	The focus groups were conducted under the surveillance of prison officers	Interview guide based on themes most commonly identified in the literature and previous surveys undertaken on the New South Whales prisoner population.
Cheng *et al*. 2018 [54], China	Psychiatric teleconsultations	2014–6	86 men in an unspecified number of correctional facilities	Survey	Not specified	Not reported	Not reported	Developed by the authors
Colombo *et al*. 2022 [20], Germany	Video consultations with specialists in general medicine and psychiatry	2018–9	20 men and 44 women in 5 correctional facilities	Interview and survey	Every patient who took part in a video consultation during the study period received a questionnaire from nursing staff. Participants were informed about the possibility of taking part in a guided interview and could inform the nursing staff if they were willing to participants.	Not reported	Transcripts were pseudonymized during transcription	Developed based on existing instruments and piloted with 7 people in 2 of the 5 participating prisons
Condon *et al*. 2007 [11], England	Healthcare services	2005	101 men and 10 women in 12 correctional facilities	Focus groups and interview	Recruited through posters instructing potential volunteers to complete a reply slip or inform prison staff of their interest. The list was vetted to exclude those who might present a risk to the physical or mental health of either the individual or the researchers	Researchers	All interviews were conducted in privacy, to the extent that healthcare or prison staff were not within listening distance of the interview	Not stated
Cuadrado *et al*. 2021 [49], Spain	Telemedicine HCV treatment	2016–7	75 people in 1 correctional facility	Survey	All people in custody experiencing a ‘test and treat’ HCV programme supported by telemedicine	Self-administered	Not reported	Not reported
Galli *et al*. 2019 [61], Swizerland	Health care services	2012–3	30 men and 5 women in 12 correctional facilities	Interview	Potential participants were initially informed about the study by medical or other prison employees. Interested prisoners then received the participant information.	Researchers	Private interviews without prison staff present	Developed by researchers
Heidari *et al*. 2017 [60], Switzerland								
Hankins *et al*. 2022 [33], UK	Healthcare services	2017	4 men and 8 women in 2 correctional facilities	Interview	Potential participants were approached while in the waiting area of the healthcare unit of the prison and provided a verbal explanation by a researcher and a participant information sheet	A prison health researcher	Participants were assured that the interview was confidential.	Not stated
Hsieh *et al*. 2022 [62], Taiwan	Healthcare services	2013.	580 men in 1 correctional facility	Survey	Not stated	Not reported	The respondents’ privacy and the data’s confidentiality were maintained in accordance with individual laws.	Developed by authors; included Patient Satisfaction Questionnaire Short Form (PSQ-18), developed by the RAND Corporation for use in community settings
Jaffer *et al*. 2016 [23], USA	Transgender Hormone Therapy	Not reported	2 transgender males and 25 transgendered females at an unreported number of correctional facilities	Verbally administered survey	Review of pharmacy records to identify the population of transgender people. Identified people were confidentially asked to participate in an anonymous survey with the jail clinic in that facility	Not reported	Patients were asked questions included in the survey in confidential clinic settings.	Developed for study informed by review of patient complaints
Jimenez-Galan *et al*. 2019 [50], Spain	Telemedicine consultation for HCV treatment	2015–8	99 men in 1 correctional facility	Survey	An anonymous questionnaire was randomly delivered to 134 participating people	Researcher	The survey was described as ‘anonymous’	Not reported
Jordan 2012 [34], UK	Primary and secondary mental healthcare services	Not reported	4 women in 1 correctional facility	Interview	Potential participants were invited to participate by their mental health clinician	Author	Transcripts had to be submitted to the Security Department and interviews were conducted in the presence of the patient’s mental healthcare clinician.	Not stated
Kanbergs *et al*. 2019 [24], USA	Healthcare services	2016	13 men and 5 women in 2 correctional facilities	Interview	All primary care practitioners working in the prison facilities were briefed on the study and asked to refer eligible individuals using a permission-to-be-contacted sheet.	Principle investigator	Participants were assured of confidentiality. Interviews were conducted without prison or medical staff present.	Developed by researchers and reviewed and approved by both the medical director of the prison system, and a human rights lawyer with extensive experience in prison health.
Karachaliou *et al*. 2024 [55], Greece	Telepsychiatry	2020–1	100 men in 2 correctional facilities	Verbally administered survey	Not reported	Not reported	Anonymous participation	Adapted from existing questionnaires developed by the Ministry of Health
Khalaf *et al*. 2019 [57], Lebanon	Treatment for opioid use disorder	2017	30 men in 1 correctional facility	Survey	Not reported	Researcher (also participants’ treating psychiatrist)	Not reported	Used tool validated for OUD treatment (not prison-specific)
Lafferty *et al*. 2022 [42], Australia	HCV RNA testing	2020–1	24 men in 1 correctional facility	Interview	After participating in the one-stop-shop, participants identified were approached by the study nurse and invited to participate	Study nurse	Only the interviewer and participant present during interview	Developed by authors
Lafferty *et al*. 2023 [43], Australia								
Liauw *et al*. 2021 [47], Canada	Reproductive health care services	2018	21 women in 1 correctional facility	Focus groups	Recruited through posters and announcements in prison common areas	A female research team member with graduate level training in qualitative methods	Only the participants and facilitator were present. Given the sensitive and personal nature of the conversations, participants were asked to respect each other’s experiences and keep what was shared in the group confidential.	Developed by project team
Magaletta *et al*. 2000 [25], USA	Pilot project of telehealth consultation with psychologist	1997–8	75 adult men in 2 correctional facilities	Survey	Not reported	Not reported	Not reported	Developed by a psychologist assigned to a US penitentiary.
Marco *et al*. 2013 [51], Spain	Methadone treatment	2010	158 men in 1 correctional facility	Survey	All study-eligible men were approached by a member of the staff in charge of their treatment and asked about their willingness to participate and provided an information sheet	A member of health care team	People were asked not to write their names on questionnaires to protect anonymity; members of healthcare team were in the room and engaged as supports.	Used instruments designed and validated to evaluate the satisfaction of patients using community health services
Mekhjian *et al*. 1999 [26], USA	Telemedicine for specialist care	Not reported	203 men and 18 women in an unspecified number of correctional facilities	Survey	Immediately after each consultation, patients were asked to complete a 14-item questionnaire	Not reported	Not reported	Developed using literature on patient satisfaction
Morgan *et al*. 2008 [27], USA	Tele- and face-to-face psychology and psychiatric services	Not reported	186 men at 1 correctional facility	Survey	People were recruited at the conclusion of a regularly scheduled appointment with psychiatry or psychology (tele or face-to-face)	Not reported	Not reported	Used tools validated in community
Motshabi *et al*. 2011 [52], South Africa	Voluntary counselling and testing for HIV	2009	200 men in 1 correctional facility	Interview and survey	Quota sampling as a convenience sample was used to ensure representation of demographic variables and an even split of those who had and had not received VCT for HIV in the correctional centre	Not reported	Not reported	Not reported
Myers *et al*. 2006 [28], USA[28]	Telepsychiatry services	2002–4	87 young men and 28 young women in 1 youth correctional facility	Survey	Participants completed a satisfaction questionnaire at the end of the evaluation	Survey distributed by correctional staff	Comments were not anonymous—staff wrote the youth’s name across the top of the questionnaire as it was handed to the youth.	Based on questionnaires described in the telemedicine literature
Plugge *et al*. 2008 [35], England	Healthcare services	Not reported	49 women in 2 correctional facilities	Focus groups and interview	Researchers identified women eligible from the local inmate directory. Researchers approached women on the wings at least 1 day before the group to given them a written information sheet and ensure they were able to attend if they wished to. Interviews used convenience sampling among women participating in a longitudinal questionnaire survey.	Researcher	No prison or healthcare staff were present in the room, and it was made clear to the women that what was said in the groups and interviews was confidential.	Not reported
Rogalska *et al*. 2022 [59 ], Poland	Healthcare services	2020	389 men and 77 women in 1 correctional facility	Survey	Not reported	Survey distributed by correctional staff	Identifiable data were not collected	Not reported
Ross *et al*. 2011 [36], UK	Healthcare services and suicide prevention work	2001–4	4379 men and 508 women in 49 correctional facilities	Survey	A sample of 100 inmates from each institution. It was not stated how potential candidates were approached	Not reported	Survey was filled out anonymously	Measuring quality of prison life
Russell *et al*. 2022 [48], Canada	Opioid agonist treatment (OAT)	2019	40 men and 6 women in 7 correctional facilities	Interview and verbally administered survey	List of eligible individuals was provided by CSC to site contacts (such as healthcare administrators). Site contacts approached individuals and provided them with study flyer which included toll-free line people could anonymously call research team if interested. Study flyer was also posted openly on the healthcare unit Individuals also had the option to express interest to study staff directly Two researchers made week-long visits to each institution to screen people who had expressed interest for eligibility.	Researchers	Participants were informed that the study was being confidentially conducted by an external research group (i.e. not affiliated with CSC) without individual study information being shared with anyone outside the research team. No personal identifying information was collected.	Not stated
Sawasdipanich *et al*. 2018 [63], Thailand	Healthcare services	Not reported	107 women in 11 correctional facilities (interviews) 405 women in 2 correctional facilities (survey) 51 women in 2 correctional facilities (focus groups)	Interview, focus groups, and survey	Not reported	Not reported	Not reported	Not reported
Schroeder & Bell 2005 [22], USA	A doula support programme	Not reported	14 women in 1 correctional facility	Interview	Asked all programme participants	Not reported	Not reported	Not stated
Sifunda *et al*. 2006 [53], South Africa	General experiences of health services	Not reported	Number of participants not described within 4 correctional facilities	Focus groups	Not reported	IsiZulu speaking facilitators	Not reported	Developed based on available literature and pre-tested in one prison in each of the two provinces.
Sindicich *et al*. 2016 [44], Australia	Opioid substitution therapy (OST)	2012–3	32 men and 15 women in 7 correctional facilities	Interview	Identified through Drug and Alcohol Database, referrals from clinical nursing staff, recruited via the reception health assessment, referrals from interviewees	Not reported	Confidentiality was assured	Not reported
Tanguay et. al 2014 [29], USA	Medical or mental health prescriber encounters, nurse encounters, dental, mental health staff, laboratory services, counselling services and/or pharmacy actions	Not reported	2469 adult men and 258 adult women in 17 correctional facilities	Survey	People who had received healthcare services that day were approached by staff from the CMHC central office	CMHC central office staff	Participants were instructed not to write their name or identification number on the survey and were encouraged to place written responses directly in a sealed collection box.	Questions derived from the health survey, the 2001 Institute of Medicine (IOM) report Crossing the Quality Chasm: A New Health System for the 21st century and existing satisfaction surveys
Tucker 2005 [37]	A pharmacist dermatology clinic	Not reported	106 men at 2 correctional facilities	Survey	All people seen at clinics were invited to complete a questionnaire at a follow-up appointment	Self-administered and submitted to pharmacist	All prisoners were assured of the anonymity and confidentiality of the information they provided. Participants sealed completed forms in an envelope and handed it back to the pharmacist before leaving	Adapted from an existing questionnaire and piloted with a sample of 10 people.
Way *et al*. 2007 [30], USA	Mental health services	2004	613 participants in 21 correctional facilities	Survey	Mental Health staff at each prison unit distributed surveys to a random sample of active patients seen in the Clinic, Disciplinary Housing, and a Residential programme for fragile seriously mentally ill patients.	Self-administered, collected by mental health staff in a sealed envelope and mailed to Central New York Psychiatric Center	Patients were asked to complete the questionnaire in private and seal it in an envelope addressed to the first author. Patient names were not collected.	The study adapted the Client Satisfaction Survey and the MHSIP Adult Survey for use in prison
Williams *et al*. 2022 [38], UK	NHS Health Checks	2017–8	25 men in 5 correctional facilities	Focus groups	People eligible to receive a health check were invited to participate	Researcher	Not reported	Not reported
Wong *et al*. 2018 [45], Australia	Two newly implemented nurse practitioner models of care	2015	29 men in 1 correctional facility	Survey	All patients were approached to complete the patient satisfaction survey after each episode of care.	Distributed by Nurse Practitioner	The questionnaire was provided to the patient to complete anonymously the end of each episode of care	Adapted from a questionnaire already used by Prison Health Services
Young 2000 [31], USA	Healthcare services	Not reported	15 women in 1 correctional facility	Interview	Mailed an introductory letter requesting participation in an interview, along with a form to submit if willing to be interviewed	Researcher	Private office, confidentiality was carefully guarded	Developed by the author
